# Artificial Intelligence-Enhanced Assessment of Lipid Profiles in Commercial Infant Foods in the United States

**Published:** 2026-02-20

**Authors:** Clement G. Yedjou, Kevine Makoudjou, Monica Ochapa, Jinwei Liu, Samia Messeha, Paul B. Tchounwou

**Affiliations:** 1 Department of Biological Sciences, College of Science and Technology, Florida Agricultural and Mechanical University, 1610 S. Martin Luther King Blvd, Tallahassee, FL 32307, USA;; 2 School of Community Health and Policy, Morgan State University, 4530 Portage Avenue Campus Near, E. Cold Spring Lane, Baltimore, MD 21251, USA;; 3 Department of Computer and Information Sciences, College of Science and Technology, Florida Agricultural and Mechanical University, 1610 S. Martin Luther King Blvd, Tallahassee, FL 32307, USA;; 4 RCMI Center for Urban Health Disparities Research and Innovation, Morgan State University, 1700 E. Cold Spring Lane, Baltimore, MD 21251, USA

**Keywords:** Infant nutrition, Lipid content, Commercial baby food, Artificial intelligence, Nutritional analysis

## Abstract

**Introduction:**

Normal consumption of lipids is essential for optimal baby growth and neurological development, yet comprehensive analysis of lipid content in commercial baby foods remains limited. This study provides the first systematic, AI-enhanced assessment of lipid profiles across commercially available infant food products.

**Methods:**

A cross-sectional analysis of 245 commercial baby food products was conducted using artificial intelligence and machine learning algorithms for data processing and pattern recognition. Among the 245 commercial baby food products in the United States, 25 products have zero lipid content, while 220 products contain lipids ranging from 0.1 to 28.58 g of lipids. Products were categorized by total lipid content per 100g into four groups: Zero (0.0g), Low (≤ 1.0g), Moderate (>1.0–5.0g), and High (>5.0g). Statistical analyses included descriptive statistics and correlation analysis between ingredient profiles and lipid content.

**Results:**

Our analysis revealed that zero-lipid foods for infants comprised 10.2% of food products and were exclusively fruit-based, naturally low in fat, and suitable for early feeding, but limited in energy input. Low-lipid foods for infants accounted for the largest dataset and represented 45.3%, consisting of vegetable purées, fruit purées, and diluted fruit formulations, which reflect typical plant-based lipid profiles yet potentially require complementary higher-fat foods to meet caloric needs. The moderate-lipid food products accounted for 33.9% of the dataset. These food products included grain-vegetable mixtures, fortified fruit blends, and enriched beverages, indicating the incorporation of added oils and/or higher-fat natural ingredients to provide balanced energy.

High-lipid foods such as fortified cereals, meat-based meals, and snack-type products made up 10% of the dataset. These items provide significant energy, which is especially important for infants between 6 months and 2 years old, as they have increased caloric needs relative to their size.

**Conclusion:**

Using AI and machine learning, this analysis revealed that baby foods on the market show clear variations in fat content linked to their main ingredients. Since many foods are high in fat, caregivers may need to make intentional choices to ensure infants receive adequate energy. These findings offer valuable reference points for parents, pediatric nutrition advice, and future regulatory decisions.

## INTRODUCTION

Lipids represent a group of fatty compounds essential for human physiology, providing long-term energy storage, regulating membrane permeability, supporting cellular insulation, and signaling pathways [1]. In early life, lipids are particularly critical because they supply essential fatty acids, promote myelination, support synaptogenesis, and contribute to the rapid brain growth that occurs during infancy and toddlerhood [2,3]. Dietary guidelines indicate that infants, especially those between 6 and 24 months of age, require a high proportion of their total caloric intake from fats typically 30%−40% to sustain neurologic development, immune function, and healthy growth trajectories [4]. Although lipids are indispensable for development, studies have revealed that excessive lipid levels in the bloodstream have been linked to long-term cardio-metabolic risks, such as cardiovascular disease, early onset of atherosclerosis, and metabolic dysfunction [5]. Studies also revealed that high intake of fats in early childhood is associated with increased risk of obesity later in life; impairs endothelial function and accelerates atherogenesis; contributes to insulin resistance, systemic inflammation, and metabolic syndrome; and disrupts hepatic lipid metabolism and insulin signaling [6–9]. In the United States, commercial baby foods make up a significant part of many infants’ diets. Despite their common use, little research has systematically examined the fat content of these products. Recent studies indicate that lipid levels vary widely by brand and food type, highlighting potential concerns about nutritional consistency and adequacy [10,11]. To date, no published research has utilized Artificial Intelligence (AI) or machine learning to conduct large-scale nutrient profiling of infant foods, even though these tools have proven valuable in nutritional epidemiology, food composition research, and diet quality assessment [12–14].

With AI/ML methods being increasingly adopted in nutrition science, automated data analysis offers new ways to classify products, uncover latent nutrient trends, and inform more precise, evidence-based feeding guidance. This study introduces the first AI-enhanced lipid profiling of U.S. commercial baby foods, examining variations in fat content across product categories and discussing the potential impacts on pediatric nutrition, dietary quality, and early-life health.

## METHODOLOGY

### Research methodology includes

#### Study design:

This cross-sectional analysis was conducted to systematically evaluate the lipid content of commercial infant food products. The design allowed for a comprehensive snapshot of lipid distribution patterns across product types at a single point in time.

#### Data source:

Data were sourced from the United States Department of Agriculture and accessed *via* a publicly available Kaggle dataset: The nutritional content of food: A comprehensive dataset.

#### Dataset:

A total of 245 commercially available baby food products were analyzed. Lipid content per serving was extracted and standardized to daily intake estimates for comparison.

#### Lipid content categorization:

Using AI-driven clustering and supervised classification (k-means and decision tree models), products were grouped into four lipid categories based on total lipid per 100 g (Table 1).

Thresholds were informed by standard infant nutrition guidelines and preliminary data distribution.

#### Selection and exclusion criteria:

All 245 identified baby food products with reported lipid content (0–28 g/100 g) were included. No products were excluded, ensuring an unbiased representation of lipid variability in the marketplace.

#### AI and machine learning workflow:

AI/ML tools were applied to:

- Automate nutrient profile extraction
- Classify lipids into four categories
- Identify ingredient-lipid associations
- Perform quality control (detect inconsistencies, duplicates, or missing entries)

#### Statistical analysis:

Analyses were conducted using Microsoft Excel (Data Analysis Tool Pak), SPSS and R statistical software.

#### Descriptive statistics:

Frequency distributions, measures of central tendency and dispersion, and AI-assisted visualization (bar charts, histograms).

#### Correlation analysis:

Pearson and Spearman tests were used to examine relationships between total lipid content and ingredient composition, as well as between product category (e.g., fruit, vegetable, or meat-based) and lipid levels. Normality assessments guided the choice of the correlation coefficient.

## RESULTS

### Lipid distribution in commercial baby foods and consistency with dietary guidelines

An evaluation of lipid content across commercial infant and baby food products revealed that zero-lipid items accounted for 10.2% of the total, consisting exclusively of fruit-based foods that are naturally low in fat. While appropriate for early feeding stages, these products provide limited energy density.

Low-lipid products made up the largest share (45.3%) and were primarily fruit purées, vegetable purées, and diluted fruit formulations. These items reflect the inherently low-fat content of most plant-based foods but may need to be paired with higher-fat options to adequately support infants’ energy needs.

Moderate-lipid products represented 33.9% of the dataset and included fruit blends, grain-vegetable combinations, and enriched beverages, suggesting the intentional inclusion of added oils or naturally higher-fat ingredients to enhance energy balance.

High-lipid products accounted for 10.6% of items and comprised meat-based meals, fortified cereals, and snack-type foods. These products deliver concentrated energy and are particularly important for infants aged 6–24 months, who have high caloric demands relative to body weight (Table 2).

**Figure 1:**
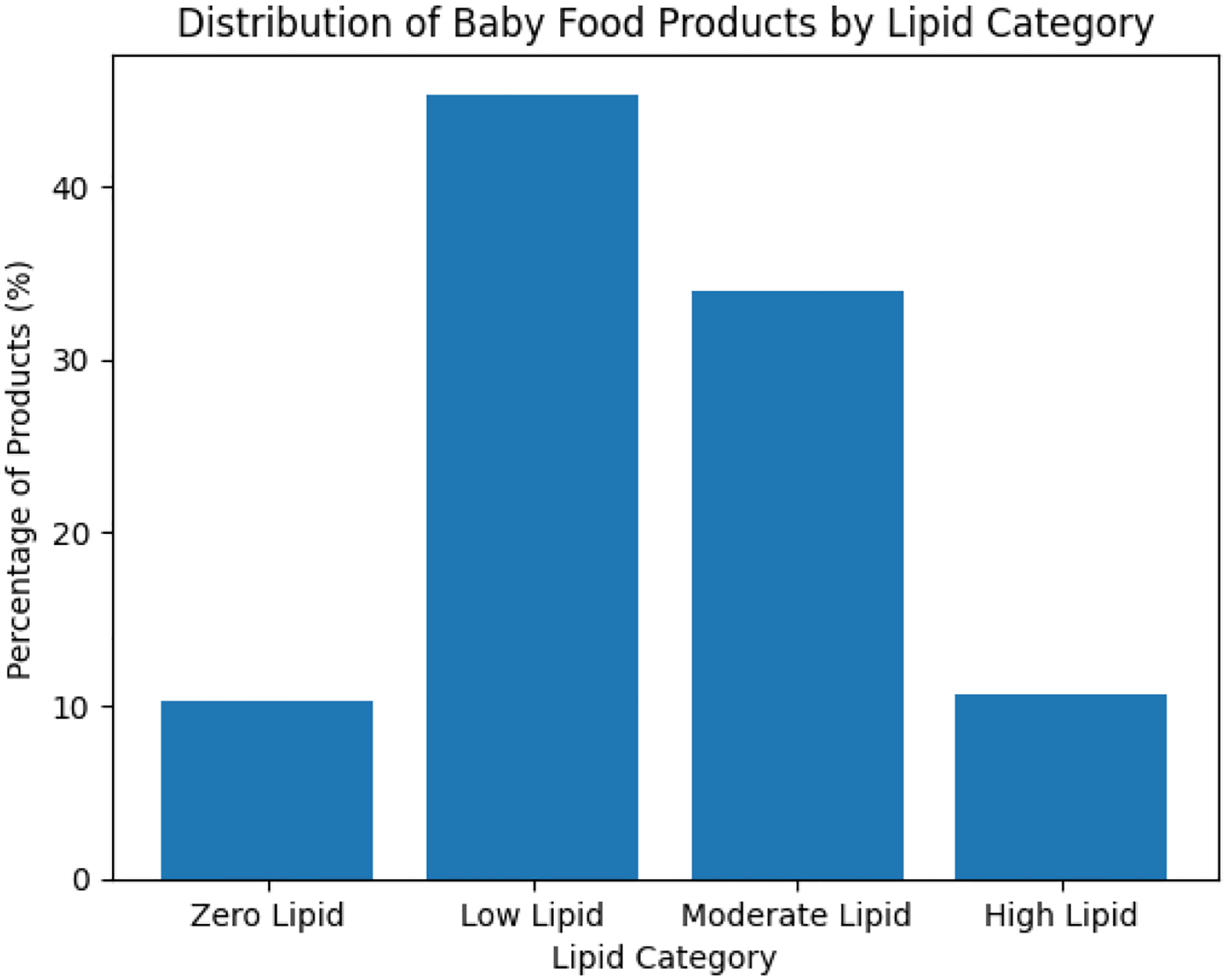
Distribution of baby food products by lipid category.

As described in Figure 1 below, this study revealed the following findings:

- The low-fat foods (0.1–1.0 g/100 g) dominate the dataset and account for 45% of items.
- The moderate-fat foods (1.1–5.0 g/100 g) have the second largest dataset, comprising approximately 34%, mostly mixed formulations and fortified blends.
- The zero-fat and high-lipid categories each contribute roughly 10%−11%, representing fruits/juices and meat-based or fortified products, respectively.

As seen in Figure 1, there is a predominance of low-and moderate-fat products supports balanced energy intake during complementary feeding, while the relatively limited content of zero- and high-lipid products reflects appropriate formulation practices that prioritize nutritional adequacy and promote healthy growth and development.

**Figure 2:**
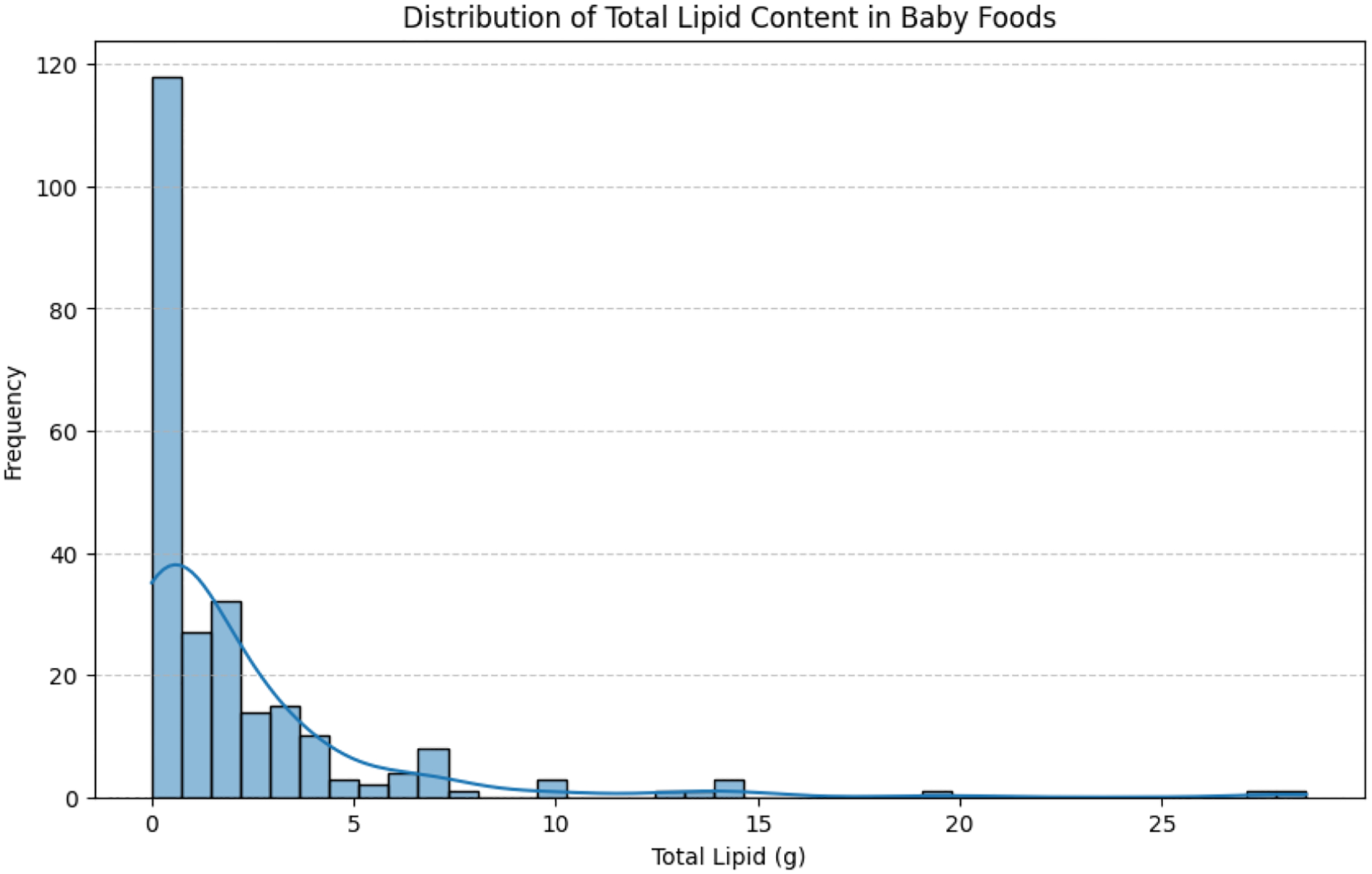
Distribution of lipid content in baby food products.

Similar to Figure 1, showing the distribution of baby foods by lipid category, we analyzed the total lipid content across our dataset. Our analysis of the total lipid content in baby foods reveals a highly right-skewed distribution, with the majority of foods containing low levels of fat, typically between 0 and 3 grams (Figure 2). This observation indicates that most baby foods are formulated to provide hydration, micronutrients, and dietary variety rather than serving as a primary source of dietary fat. A smaller subset of baby foods exhibits moderate to high lipid content, likely reflecting processed snack foods and meat-based items with added or naturally occurring fats. These high-lipid outliers contribute substantially to overall variability in the dataset.

### Alignment of Figure 1 lipid categories with infant nutrition guidelines

Table 3 summarizes the distribution of lipid categories in commercially available baby foods in the United States and their alignment with dietary recommendations from the World Health Organization (WHO) and the American Academy of Pediatrics (AAP).

### Mapping lipid categories to World Health Organization (WHO) and American Academy of Pediatrics (AAP) fat intake recommendations

Dietary fat intake is important during infancy and early childhood to support rapid growth, brain development, and metabolic function. According to guidelines from the World Health Organization (WHO) and the American Academy of Pediatrics (AAP), infants and young children typically require approximately 600–750 kcal per day, with a substantial proportion of total energy derived from fat. The percentage of calories contributed by fat decreases gradually with age as dietary patterns diversify. Table 4 summarizes the estimated recommended daily fat intake across developmental stages, calculated based on age-specific energy requirements and the proportion of total calories recommended to come from fat.

During the first six months of baby’s life, dietary fat intake is often highest, reflecting reliance on the mother’s breast milk and/or infant formula as primary energy sources. As complementary foods are introduced between 6 and 12 months old, the proportion of dietary fat decreases gradually while remaining sufficient to support neurodevelopment. By 1–3 years of age, fat intake stabilizes at approximately 30%−40% of total energy, consistent with WHO and AAP recommendations emphasizing balanced energy intake and nutrient diversity.

The diagram in Figure 2 presents a four-quadrant visual comparison of baby food products categorized by lipid content: Zero-lipid, low-lipid, moderate-lipid, and high-lipid foods. Each category is clearly labeled and illustrated with representative food images.

#### Zero-lipid products:

**Figure 3:**
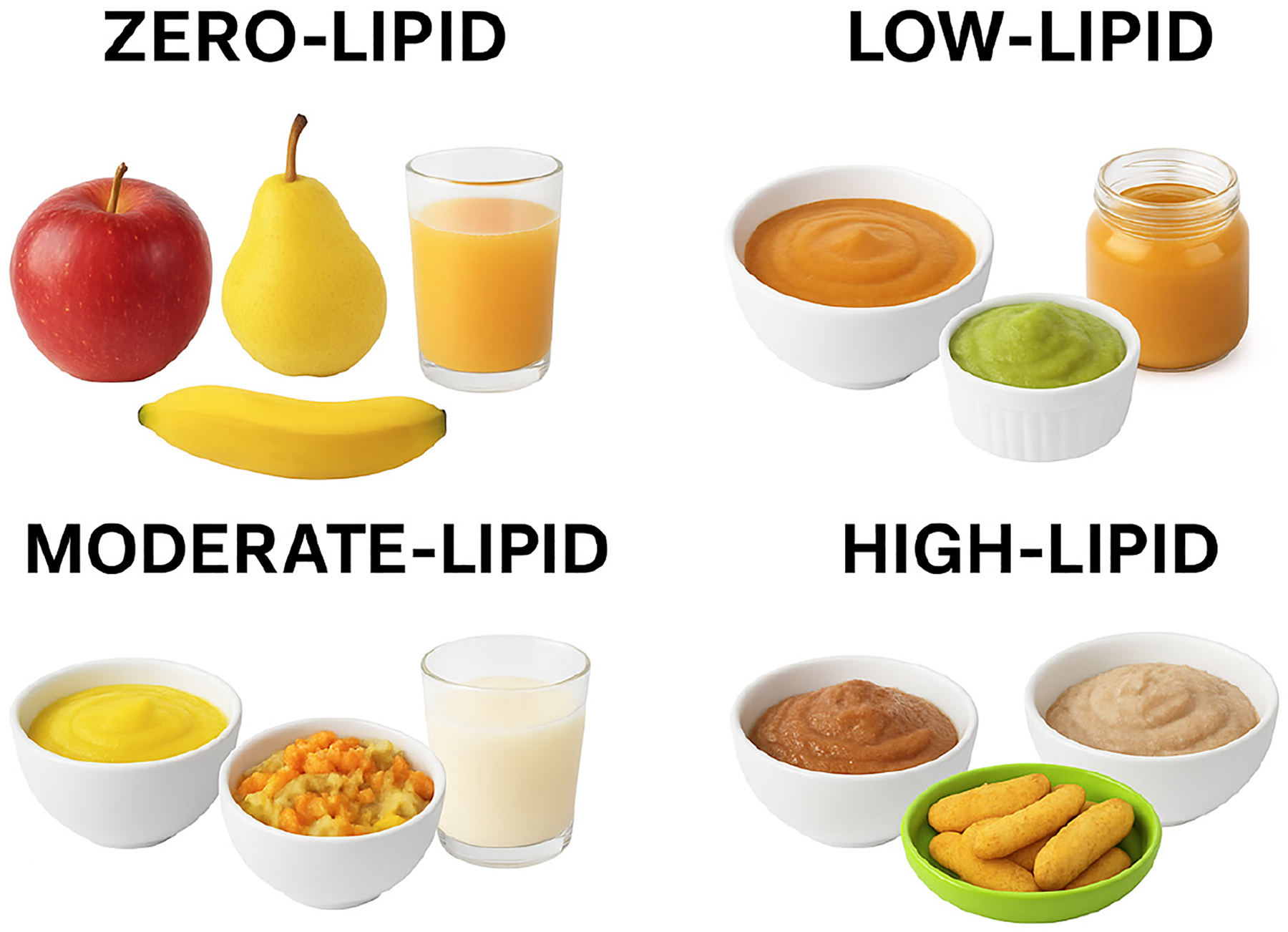
AI-generated images showing lipid content in baby foods range from zero to high lipid levels. It visually compares meals containing zero, low, moderate and high lipid content.

These non-fat items, illustrated in Figure 3, are mostly whole fruits and fruit-based beverages, including a banana, an apple, a pear, and a glass of fruit juice. These zero-lipid products contain negligible fat content, are typically rich in water, natural sugars, and other micronutrients, and are commonly beneficial to promote hydration and vitamin intake rather than energy provision.

#### Low-lipid products:

These low-fat items, represented in Figure 3, feature smooth fruit and vegetable purées presented in bowls and jars. These products are lightly processed and provide a good, small amount of fat, and are used during the early stage of complementary feeding to introduce new tastes, flavors, and textures.

#### Moderate-lipid products:

The images of the moderate-lipid food quadrant include fortified purées, mixed vegetable-grain blends, and an enriched beverage. The foods shown have a moderate fat content, which supports gradual increases in dietary fat intake necessary for brain development, myelination, and metabolic processes during later stages of infancy.

#### High-lipid products:

These high-fat foods include products such as thicker cereal blends, meat-based purées, and finger-style snacks. These foods are more energy-dense and have a higher fat content, making them suitable for older infants and toddlers when consumed in appropriate portions to support growth and meet increased energy demands. Overall, the visualization in Figure 2 reinforces the gradual increase in fat content in infant foods and illustrates how different food types contribute distinct nutritional roles within infant and young child diets.

**Figure 4:**
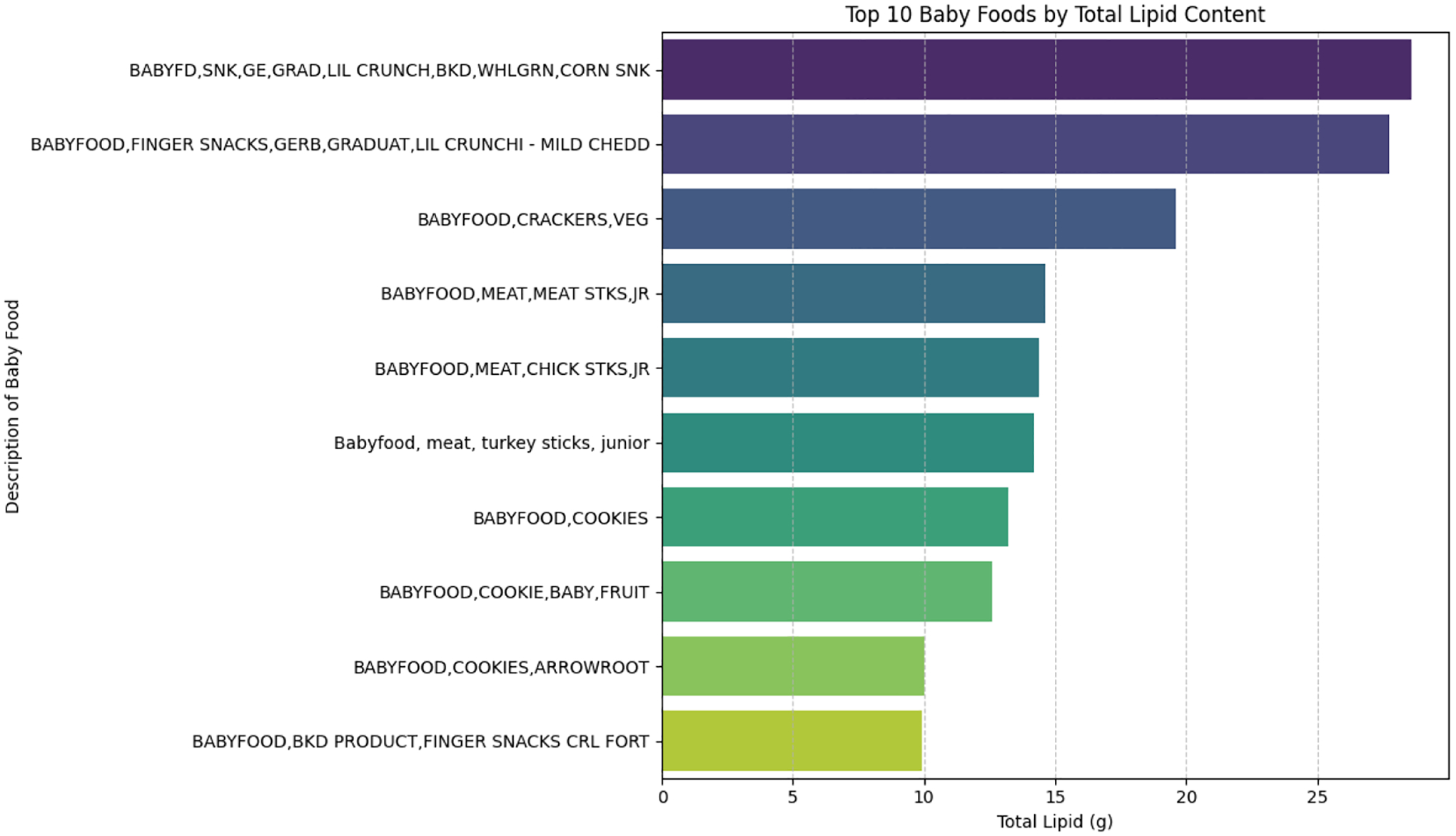
Top ten (10) baby foods products by total lipid content.

Figure 4 below shows a horizontal bar chart, in which baby food items are ranked according to their total lipid or fat content expressed in grams (g). As illustrated in this figure, the x-axis represents total lipid content (g), while the y-axis lists descriptions of baby food items. It demonstrates substantial variability in lipid content across commonly consumed baby foods in the United States, with processed snack products contributing the highest fat levels.

### Correlation analysis of fat categories in baby food products

In this study, we performed a correlation analysis to explore the relationship between fat content categories and product distribution in commercially available baby foods. Lipid content was treated as an ordinal variable (0=zero lipid, 1=low lipid, 2=moderate lipid, and 3=high lipid), while product prevalence was assessed using the percentage of products within each category.

Table 5 reveals a correlation coefficient (r=−0.075), indicating a negligible negative linear relationship between lipid category and product prevalence. This low correlation value reflects the fact that the association between these variables is really nonlinear. Interestingly, the visual inspection of the distribution reveals a nonlinear, inverted U-shaped pattern, in which product prevalence increases from the zero-lipid category (10.2%) to the low-lipid category (45.3%), remains relatively high in the moderate-lipid category (33.9%), and then declines sharply in the high-lipid category (10.6%). The highlighted observation reveals that infant foods are commonly formulated with low to moderate fat content, rather than high fat content.

These results indicate that moderate fat levels represent an optimal range, which balances nutritional requirements with formulation practices. This distribution is consistent with infant and young child feeding recommendations that emphasize adequate, but not excessive, dietary fat intake to support growth and neurodevelopment.

## DISCUSSION

Analysis of lipid content across infant and baby food products shows a clear predominance of low-lipid formulations (45.3%), followed by moderate-lipid products (33.9%). Zero-lipid and high-lipid categories represented approximately one-tenth of total products (10.2 and 10.6%, respectively). The zero-lipid foods observed in this study align with recommendations that these items should play a supportive role and not replace fat-containing foods necessary for optimal brain development [15]. Low-lipid foods consisted mainly of fruit and vegetable purees and water-based formulations, while moderate-lipid foods were largely fortified blends and mixed food matrices. High-lipid items were primarily meat-based products, fortified cereals, and snack-type formulations. The small share of high-lipid products is consistent with guidance that does not encourage high intake of energy-dense foods that may increase the risk of adverse metabolic outcomes [16,17]. Overall, this distribution aligns with dietary guidance from the World Health Organization (WHO), the American Academy of Pediatrics (AAP), and the Institute of Medicine (IOM), all of which emphasize the critical importance of dietary fat for early growth and neurodevelopment [18,19]

It has been advised to secure about 40%−55% of babies’ total energy from fat, largely through breast milk or baby formula, during the first six months of life [16,19]. Foods introduced after six months of age are not intended to serve as the main source of dietary fat. As a result, the high percentage of low-lipid products tested in this analysis aligns with infant feeding recommendations, as these foods mainly provide essential micronutrients, dietary diversity, and hydration, while the majority of fat requirements continue to be supplied through breast milk or formula.

Between 6 and 24 months of age, the World Health Organization (WHO) and American Academy of Pediatrics (AAP) guidelines recommend a progressive reduction in fat consumption to about 30%−40% of total energy, with greater emphasis on fat quality rather than quantity [20]. The substantial percentage of moderate-lipid products in the dataset supports this developmental transition and reflects appropriate formulation approaches designed to meet evolving metabolic and neurological requirements.

## CONCLUSION

The current findings demonstrate that the relatively small percentage of zero-lipid products, mainly fruits and juices, supports the guidance that these items should play a complementary rather than primary role in meeting energy needs, particularly after 6 months of age. On the other hand, the low level of high-lipid food products aligns with dietary recommendations that caution against high energy in childhood diets, as this may displace key nutrients, increasing the risk factors and unfavorable or ‘adverse metabolic outcomes. The majority of low- and moderate-lipid food products are appropriate for the formulation strategies that support complementary feeding by delivering essential micronutrients and balanced energy while allowing breast milk to remain the primary source of dietary fat in early infancy.

In summary, our evaluation of lipid content in commercial baby foods in the marketplace in the United States indicates a distribution that is mostly consistent with established young child nutrition guidelines issued by the American Academy of Pediatrics (AAP), the Institute of Medicine (IOM), and the World Health Organization (WHO). The results generated in this research work highlight the importance of balanced fat content in baby food items and promote a practical framework for assessing commercial baby foods against internationally recognized nutritional standards.

## Figures and Tables

**Table 1:** Lipid content categorization.

Category	Count	Range (per 100g)
Zero lipid	25	0.0 g
Low lipid	111	0.1–1.0 g
Moderate lipid	83	1.1–5.0 g
High lipid	26	5.1–28.0 g

**Table 2:** Lipid content in baby food and nutritional significance.

Lipid category	Range (g/100 g)	Count (%)	Primary product types	Nutritional significance
Zero lipid	0.0–0.0 g	25 (10.2%)	Fruits (pear, banana, apple, juice, cherry and plum).	Naturally fat-free and appropriate for early feeding stages; however, these foods provide low energy density and may need to be paired with higher-fat options for infants with increased caloric demands.
Low lipid	0.1–1.0 g	111 (45.3%)	Fruit purées, vegetable purées, diluted fruit juices, water-based formulations	Physiologically appropriate for plant-based foods, reflecting the inherent lipid content of fruits and vegetables (0.1–1.0 g/100 g). At the same time, suitable supplementation is necessary to achieve sufficient energy density.
Moderate lipid	1.1–5.0g	83 (33.9%)	Fortified fruit blends, mixed vegetable-grain combinations, enriched beverages	Nutritionally balanced for mixed or combination foods, indicating the inclusion of added oils, dairy components, or naturally higher-fat ingredients that support moderate energy needs.
High lipid	5.1–28.0g	26 (10.6%)	Meat-based products, fortified cereals and snacks	Energy-dense products play a critical role in meeting the caloric requirements of growing infants and are particularly important for those aged 6–24 months, who have higher energy needs per kilogram of body weight.
Total food items		245 (100%)		

**Table 3:** Lipid categories in baby foods and their alignment with dietary recommendations.

Lipid category	Range (g/100 g)	Guideline-based interpretation	Guideline alignment
Zero-lipid products (10.2%)	0.0–0.0	The World Health Organization (WHO) and the American Academy of Pediatrics (AAP) recommend that fat-free foods should not dominate the infant diet, particularly after 6 months of age. The free fat foods (fruits and juices) in this group are intended to improve the micronutrient intake and hydration rather than energy provision. The limited representation observed in this dataset aligns with guidance that zero-lipid foods should serve a supplementary, not primary, role in infant feeding	Acceptable at a low proportion
Low-lipid products (45.3%)	0.1–1.0	The foods in this group are not designed to meet total fat requirements solely, however, complement breast milk or infant formula, which remain the primary fat sources during early infancy. Their prevalence reflects WHO and AAP recommendations that complementary foods provide vitamins, minerals and carbohydrates while supporting rather than replacing milk-based fat intake in infants under 12 months.	Strong
Moderate-lipid products (33.9%)	1.1–5.0	Moderate-lipid foods support the gradual dietary transition toward 30%–40% of total energy from fat during complementary feeding. This category provides essential fatty components required for myelination, cognitive development and hormone synthesis and closely aligns with WHO and AAP recommendations for infants and young children aged 6–24 months.	Optimal
High-lipid products (10.6%)	5.1–28.0	These foods are appropriate when consumed in small amounts, particularly meat-based products and fortified, nutrient-dense formulations. The relatively low proportion observed aligns with AAP guidance to avoid excessive energy content and prevent displacement of other essential nutrients during early development.	Appropriate but controlled

**Table 4:** Estimated fat grams per day.

Age	% of Calories from fat	Approx. fat garms/day
0–6 months	40%–55%	~30–40 g/day
7–12 months	35%–45%	~27–35 g/day
1–3 years	30%–40%	30–40 g/day

**Table 5:** Correlation analysis between fat category and product prevalence.

Variable	Ordinal value	Ordinal value
Ordinal value	1.00	
Percentage (%)	−0.075	1.00

## Data Availability

The data that support the present review article are included in the article.
